# Effects of Vitamin A on Growth Performance, Antioxidants, Gut Inflammation, and Microbes in Weaned Piglets

**DOI:** 10.3390/antiox12122049

**Published:** 2023-11-27

**Authors:** Shengnan Wu, Li Wang, Bailei Cui, Xiaolu Wen, Zongyong Jiang, Shenglan Hu

**Affiliations:** 1 Institute of Animal Science, Guangdong Academy of Agricultural Sciences, Guangzhou 510640, Chinawangli1@gdaas.cn (L.W.);; 2 State Key Laboratory of Livestock and Poultry Breeding, Guangzhou 510640, China; 3 Key Laboratory of Animal Nutrition and Feed Science in South China, Ministry of Agriculture and Rural Affairs, Guangzhou 510640, China; 4 Guangdong Key Laboratory of Animal Breeding and Nutrition, Guangzhou 510640, China; 5 Lingnan Modern Agricultural Science and Technology Guangdong Provincial Laboratory Maoming Branch, Guangzhou 510640, China

**Keywords:** vitamin A, antioxidant, weaned piglets, growth performance, gut microbiota, inflammation

## Abstract

Piglet weaning is an important stage in production where changes in the environment and diet can cause problems such as intestinal inflammation and diarrhea. Vitamin A is an essential nutrient for human and animal growth and has immunomodulatory and inflammatory effects. A large body of literature has previously reported on the use of vitamin A in piglet production, so our experiment added different concentrations of vitamin A (0, 1100, 2200, 4400, 8800, and 17,600 IU/kg) to weaned piglet diets to study the effects of different doses on growth performance, intestinal barrier, inflammation, and flora in weaned piglets. We selected 4400 IU/kg as the optimum concentration of vitamin A in relation to average daily weight gain, feed intake, feed-to-weight ratio, and diarrhea rate, and subsequently tested the inflammatory factors, immunoglobulin content, antioxidant levels, and intestinal flora of weaned piglets. Results: We observed that the diarrhea rate of weaned piglets was significantly lower after the addition of 4400 IU/kg of vitamin A to the diet (*p* < 0.05). A control group and a 4400 IU/kg VA group were selected for subsequent experiments. We found that after the addition of vitamin A, the serum CAT level of weaned piglets increased significantly, the expression of *Claudin-1* in the jejunum and ileum increased significantly, the expression of *Occludin* gene in the jejunum increased significantly, the expression of *IL-5* and *IL-10* in the ileum increased significantly (*p* < 0.05), and the expression of *IL-4*, *IL-5*, and *IL-10* in the ileum increased significantly (*p* < 0.05). Meanwhile, in the colonic flora of vitamin A-added weaned piglets, the relative abundance of *Actinobacteria* and *Erysipelotrichales* decreased significantly, while the relative abundance of *Bacteroidales* increased significantly (*p* < 0.05). The results of this study indicated that vitamin A at 4400 IU/kg reduces diarrhea in weaned piglets by increasing antioxidant levels, increasing intestinal tight junction protein gene expression, and regulating colonic gut microbiota.

## 1. Background

Weaning stress always poses a great threat to pig production, and piglets suffer from intestinal inflammation, diarrhea, oxidative stress, and flora imbalance [1,2,3]. The first line of defense against toxins and pathogenic microorganisms in the intestinal lumen, as well as antigenic substances in the feed, is the intestinal epithelial barrier (physical barrier). Cytokines play a critical role in the integrity of the intestinal barrier and are an important part of the regulation of intestinal immune barrier function. Pro-inflammatory cytokines frequently disrupt tight junction (TJ) proteins, whereas anti-inflammatory cytokines protect epithelial barrier function [4]. For animal health, the intestinal microbiota is an important environmental factor. A healthy gut microbiota contributes to the host’s nutrient metabolism, the structural integrity of the gut mucosal barrier, immune regulation, cognitive and behavioral development, and pathogen protection [5]. Previous studies have shown that dietary antibiotics can effectively solve the problems caused by stress in piglets, while the prohibition of antibiotics leads to frequent bacterial diseases, reducing pig performance, greatly increasing the rate of diarrhea, increasing the utilization rate of therapeutic antibiotics, and increasing the production cost of pigs [6]. These studies were concerned with the role of nutrients in weaning piglets.

Vitamin A is a fat-soluble compound that cannot be synthesized in animals and needs to be obtained from feed. Vitamin A, as an organic compound, can be metabolized and transported into retinol, retinoic acid, retinaldehyde, and other substances in the body by related enzymes [7,8,9,10]. Previous studies have shown that vitamin A can improve the growth performance, and antioxidant and immune capacity of animals [11,12,13]. Vitamin A deficiency lowers immunity and causes inflammation, while excessive supplementation can be toxic, causing headaches, bone pain, and brain edema [14]. Zhou et al. found that weaned piglets supplemented with vitamin A significantly improved growth performance, glutathione peroxidase levels, and serum IgA levels [11]. Hu et al. found that vitamin A enhances the activity of immunoglobulin M and glutathione peroxidase in weaner piglets by using starch and gelatin coatings [15]. Moreover, previous research has discovered that vitamin A regulates the interaction of eukaryotic host cells with symbiotic microorganisms as well as the complexity of the microbiome, which in turn regulates vitamin A metabolism in the host [16]. Therefore, the aim of this study was to investigate the effects of dietary supplementation with vitamin A on diarrhea, antioxidant capacity, immunity, and gut microbiota in piglets where no antibiotics were used, and to explore the optimum concentration of vitamin A in the diet of weaned piglets.

## 2. Methods and Materials

The experimental procedures used in this study were approved by the Animal Care and Use Committee of the Guangdong Academy of Agricultural Sciences (Guangzhou, China) (No. GAASIAS-2020-005).

### 2.1. Animal Experimental Treatments

Seventy-two piglets (Duroc × Landrace × Yorkshire) at 8.23 ± 0.10 kg average body weight were weaned at the age of 21 days and randomly divided into six groups. Piglets were randomly divided into 6 groups according to body weight, with half male and half female in each group. The six groups were the control group with basic diet and basic diet added with 1100, 2200, 4400, 8800, and 17,600 IU/kg of vitamin A, respectively, each group had 6 replicate pens per group and 2 piglets in each pen. The composition of the basic diet (Table 1) was designed according to the nutritional requirements of the National Research Council (NRC, 2012) for piglets weighing 7 to 11 kg. And no antibiotics were used in the diet. The piglets were free to feed and water throughout the 28-day experimental period. Illness, diarrhea, or any abnormal behavior was observed and recorded (Diarrhea rate% = Number of piglets with diarrhea in the trial period/(number of test heads × test time) × 100%). On days 0, 12, and 28, piglets BW and feed consumed were weighed to determine the average daily feed intake (ADFI) and average daily gain (ADG). The feed conversion ratio (F/G) of weaned piglets was calculated as the ratio of ADFI to ADG.

### 2.2. Sample Collection

Each piglet was weighed and recorded on the final day of the experiment after 12 h fasting. Blood samples were taken from the anterior vena cava of each piglet. And then, the piglet was euthanized with Zoletil 50 (Virbac Philippines, 30 mg/kg) injection. After collecting jejunal and ileal bowel segment samples and colon contents in liquid nitrogen, all samples were frozen and stored in a −80 °C ultra-freezer.

### 2.3. Antioxidant Reagent

The activities of GSH-Px, T-SOD, CAT, and T-AOC were analyzed using commercial test kits (Nanjing Jiancheng, China) according to the manufacturer’s manual and determined by a multifunctional microplate reader (SYNERGY H1, Bio Tek, Winooski, VT, USA). The activity of GSH-PX was analyzed by the colorimetric method of 5,5′-dithio-bis p-nitrobenzoic acid, and the change in absorbance at 412 nm was read. The activity of T-SOD was measured by non-enzymatic NBT test, and the change in absorbance at 450 nm was read. The activity of CAT was determined by ammonium molybdate colorimetry and the change in absorbance was read at 405 nm. T-AOC was measured according to the principle of ABST oxidation into ABST+, and the absorbance change was read at 405 nm.

### 2.4. ELISA

Immunoglobulin A (IgA), immunoglobulin G (IgG), and immunoglobulin M (IgM) in serum were determined by using enzyme-linked immunosorbent assay kits (MLbio, Shanghai, China). The procedures were strictly followed with the instructions from the kits. A microplate reader (SYNERGY H1, Bio Tek, USA) was used to analyze the samples.

### 2.5. Quantitative Real-Time Polymerase Chain Reaction

Intestine RNA was extracted using Trizol reagent (Aldlab, Shanghai, China) as described by Feng et al. [17]. The concentration and purity of RNA were determined by a nucleic acid protein detector (Ultraviolet spectrophotometer type 1000, Thermo Fisher, Waltham, MA, USA). RNA samples were used to obtain cDNA by using PrimeScript™ RT reagent Kit with gDNA Eraser Perfect Real Time RR047A kit (TaKaRa, USA). And then, cDNA was stored at −20 °C for further use. Real-time quantitative PCR instrument (CFX 96 touch Real-time fluorescence quantitative PCR System, Bio-RAD Laboratories, Hercules, CA, USA) was used to check the relative mRNAs’ expression. The PCR reaction System and reaction conditions were followed according to Lang et al. [18]. The sequences of all primers are listed below (Table 2).

### 2.6. DNA Extraction and Illumina Miseq Sequencing

Microbial DNA was extracted from fecal samples (Omega, USA) in accordance with the manufacturer’s method. The V4–V5 region of bacterial 16S ribosomal RNA gene was amplified by PCR using primers 515F 5′-barcode-GTGCCAGCMGCCGCGG)-3′ and 907R 5′-CCGTCAATTCMTTTRAGTTT-3′. The bar code is a unique sequence of six bases for each sample. PCR reaction was carried out in three 20 μL mixtures containing 4 μL 5 × FastPfu buffer, 2 μL 2.5 mM dNTP, 0.8 μL each primer (5 μM), 0.4 μL FastPfu polymerase, and 10 ng template DNA. According to the manufacturer’s instructions, amplicon was extracted from 2% agarose gel and purified using the AxyPrep DNA Gel Extraction kit (Axygen Biosciences, USA). Illumina MiSeq 250PE sequencing was performed by Shanghai Lingen Biological.

### 2.7. Statistical Analysis

All data were analyzed by SPSS 22.0 software (IBM Corporation, USA). Growth performance was analyzed by one-way ANOVA, and other experimental results were compared by *t*-test. Data are represented in tables and graphs as mean and standard error (SEM) (*n* = 6). *p* values < 0.05 were considered statistically significant, while *p* values < 0.10 were used to indicate a trend.

## 3. Results

### 3.1. Growth Performance and Diarrhea Rate

Compared to those in the control group, there was no significant difference in body weight, ADFI, ADG, and F:G after weaned piglets treated with different concentrations of vitamin A. However, the vitamin A supplementation group showed a much lower diarrhea rate than that in the control group (Table 3). After the antioxidant level, intestinal tight junction protein, and cytokine expression of all treatments were tested, the 4400 IU/kg group had more positive effects in weaned piglets than other treatments (Figures S1–S3). Therefore, 4400 IU/kg vitamin A supplemental treatment was fully chosen and discussed below.

### 3.2. Serum Antioxidant

**Figure 1:**
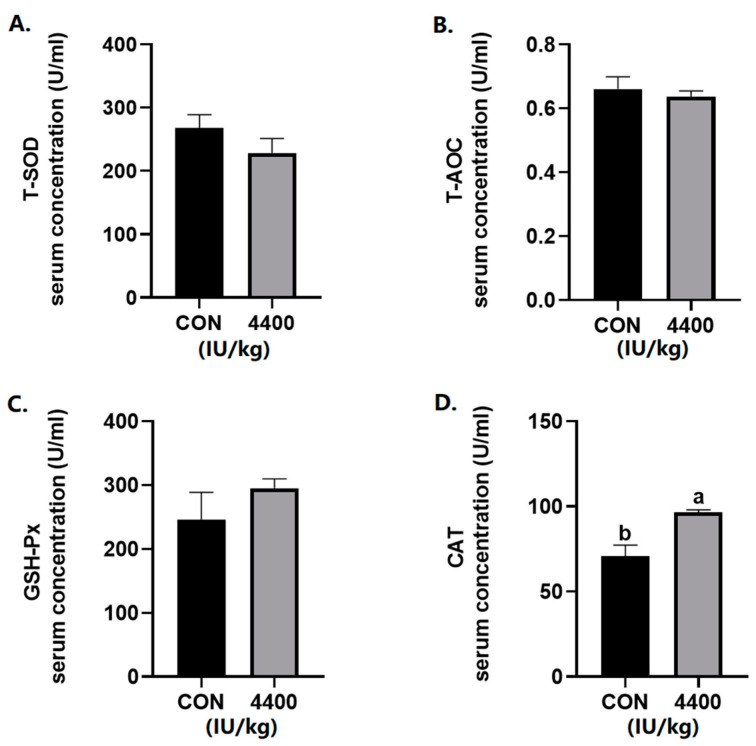
Effect of vitamin A supplementation on serum antioxidant levels: (**A**) The concentration of T-SOD in serum. (**B**) The concentration of T-AOC in serum. (**C**) The concentration of GSH-Px in serum. (**D**) The concentration of CAT in serum. a, b showed means ± SEM (*n* = 6), different letters represented significant differences (*p* < 0.05). The same as below.

According to the serum antioxidant results (Figure 1), there was no significant change in the levels of T-AOC, T-SOD, and GSH-Px. The serum CAT content was significantly increased after the addition of vitamin A (*p* < 0.05).

### 3.3. Serum Immunoglobulin

**Figure 2:**
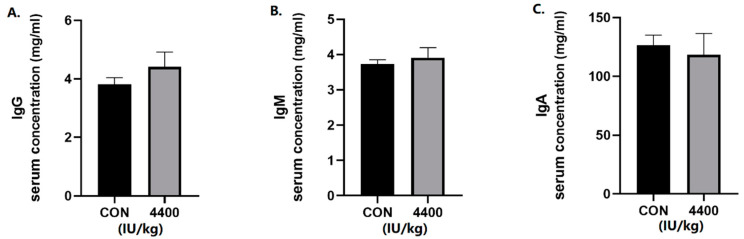
Effect of vitamin A supplementation on serum immunoglobulin levels: (**A**) The concentration of IgG in serum. (**B**) The concentration of IgM in serum. (**C**)The concentration of IgA in serum.

We further tested the serum for immunoglobulins. The concentration of IgG (Figure 2A), IgM (Figure 2B), and IgA (Figure 2C) in serum was not significantly affected by VA in the 4400 IU/kg group compared with the control group.

### 3.4. Intestinal Tight Junction Protein

**Figure 3:**
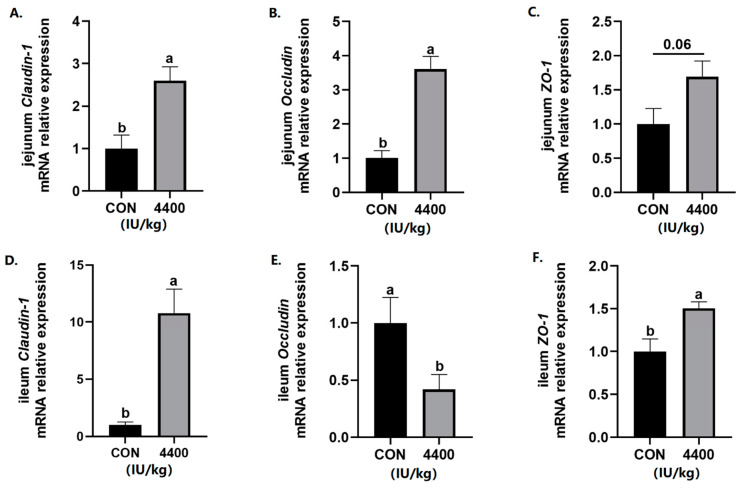
Effect of vitamin A supplementation on the expression levels of tight junction proteins in the jejunum: (**A**) Relative expression of *Claudin-1* mRNA in the jejunum. (**B**) Relative expression of *Occludin* mRNA in the jejunum. (**C**) Relative expression of *ZO-1* mRNA in the jejunum. (**D**) Relative expression of *Claudin-1* mRNA in the ileum. (**E**) Relative expression of *Occludin* mRNA in the ileum. (**F**) Relative expression of *ZO-1* mRNA in the ileum. a, b showed means ± SEM (*n* = 6), different letters represented significant differences (*p* < 0.05).

Since intestinal integrity is closely related to the diarrhea rate, intestinal tight junction protein gene expression was further detected. The data showed that compared with the control group, the expression of *Claudin-1* and *Occludin* was significantly improved in the jejunum (*p* < 0.05), and *ZO-1* expression had a trend to increase (*p* < 0.1) after vitamin A supplementation (Figure 3A–C). In the ileum, both *Claudin-1* and *ZO-1* expression was higher than the control group (*p* < 0.05), while the expression of *Occludin* was significantly decreased in the 4400 IU/kg group (*p* < 0.05, Figure 3D–F).

### 3.5. Intestinal Inflammatory Cytokines

**Figure 4:**
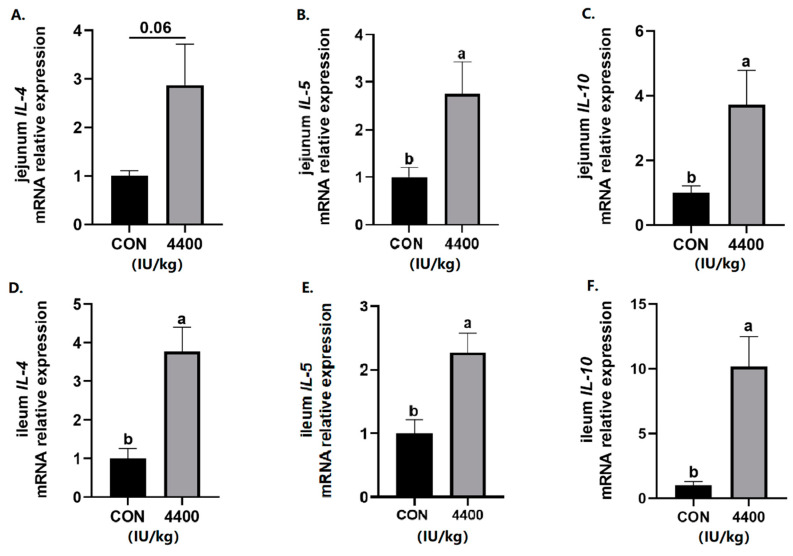
Effect of vitamin A supplementation on the expression levels of inflammatory cytokines in the jejunum and ileum: (**A**) Relative expression of *IL-4* mRNA in the jejunum. (**B**) Relative expression of *IL-5* mRNA in the jejunum. (**C**) Relative expression of *IL-6* mRNA in the jejunum. (**D**) Relative expression of *IL-4* mRNA in the ileum. (**E**) Relative expression of *IL-5* mRNA in the ileum. (**F**) Relative expression of *IL-6* mRNA in the ileum. a, b showed means ± SEM (*n* = 6), different letters represented significant differences (*p* < 0.05).

Next, intestinal cytokines expression was tested. In the jejunum, the expression of *IL-5* and *IL-10* was significantly higher (*p* < 0.05) and *IL-4* level had a rising trend (*p* < 0.1) after vitamin A supplementation (Figure 4A–C). Compared with the control group, vitamin A treatment showed a significant increase in the expression of *IL-4*, *IL-5*, and *IL-10* in the ileum (*p* < 0.05, Figure 4D–F).

### 3.6. Intestinal Contents Flora

**Figure 5:**
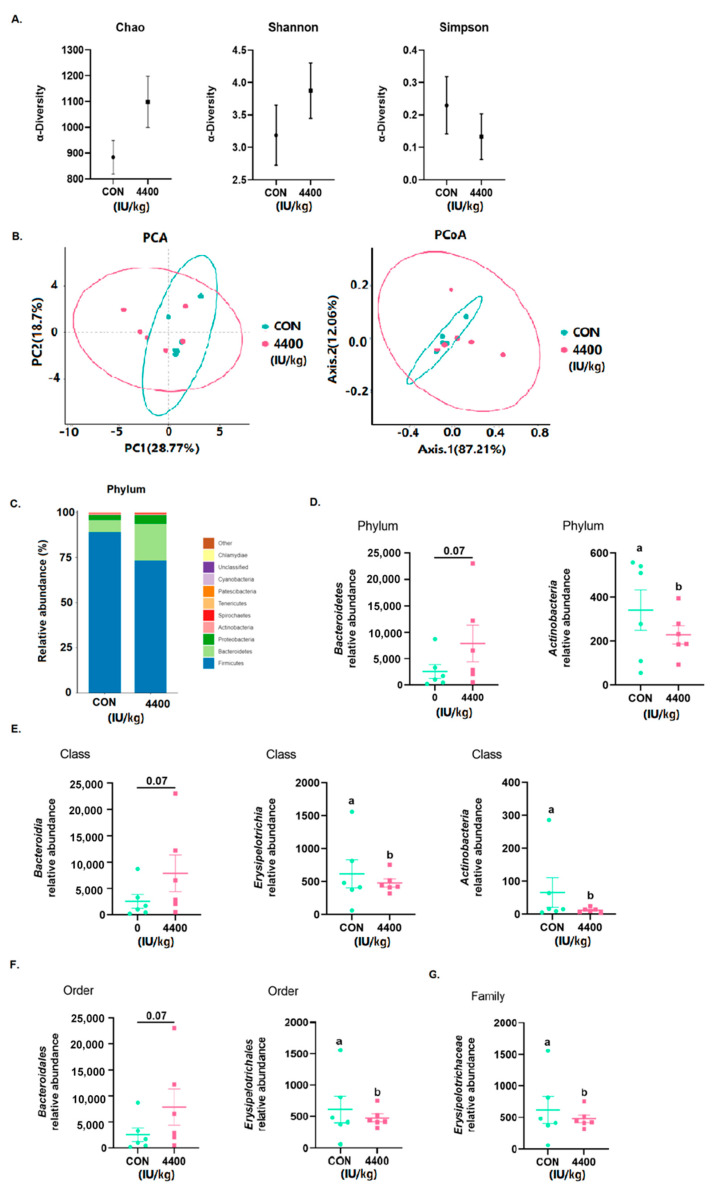
Effect of vitamin A supplementation on intestinal flora: (**A**) Alpha diversity of the cecum flora. (**B**) Beta diversity of the cecum flora. (**C**) Relative abundance of the top 20 cecal microbiota. (**D**) Bacteria with significant differences at phylum level. (**E**) Bacteria with significant differences at class level. (**F**) Bacteria with significant differences at order level. (**G**) Bacteria with significant differences at family level. a, b showed means ± SEM (*n* = 6), different letters represented significant differences (*p* < 0.05).

Using 16S rRNA Illumina MiSeq sequencing, the microbial composition of the cecum contents was determined. According to Figure 5A, no changes were found in Chao, Shannon, and Simpson. Using OTU data, we then examined the beta diversity of the cecum flora. After the addition of vitamin A (n = 6), neither PCA nor PCoA was completely overlapped with the control group (Figure 5B). *Firmicutes*, *Bacteroidetes*, and *Proteobacteria* were the three most abundant microorganisms in both groups (Figure 5C), with *Bacteroidetes* being significantly more abundant in the 4400 IU/kg group (*p* < 0.05). Finally, at the phylum (Figure 5D), class (Figure 5E), order (Figure 5F), and family (Figure 5G) levels, we examined the relative abundance of the top ten most abundant groups. *Actinobacteria* was significantly lower (*p* < 0.05) at the phylum level, while *Bacteroidetes* showed an increasing trend. *Erysipelotrichia* and *Actinobacteria* were significantly lower (*p* < 0.05) at the phylum level, while *Bacteroidetes* showed a rising trend. *Erysipelotrichales* was significantly lower (*p* < 0.05) at the order level, while *Bacteroidales* was increasing. *Erysipelotrichaceae* was significantly lower (*p* < 0.05) at the family level (Figure 5G).

## 4. Discussion

Piglets with underdeveloped intestinal function at weaning are susceptible to factors such as internal and external environment, resulting in loss of appetite, slow growth, and diarrhea. Vitamin A, as an essential microelement for animal growth, plays a positive role in growth and development and enhances immunity. Wang et al. [19] found that the supplementation of 16 mg/kg vitamin A to the diet significantly increased ADG in weaned piglets at 3 weeks of age. Our study found that vitamin A had no significant effect on body weight, feed intake, and feed/gain of weaned piglets, but vitamin A could reduce the diarrhea rate of piglets. This may be a meaningful indicator of weaning stress.

Weaning causes oxidative stress [20]. Oxidative damage is an important mechanism that damages the immune function and health of the body. The antioxidant system of the body has limited ability to clear free radicals, and excessive oxygen free radicals will destroy fat, protein, and nucleic acids in the body. Changes in the activities of oxidase GSH-Px, SOD, and CAT are closely related to the stress response of animals and can be used to reflect the immune state of the body [21]. Serum antioxidant capacity can reflect the resistance of animals to endogenous oxidative damage, and a higher antioxidant capacity has a beneficial effect on alleviating oxidative stress [22]. CAT is an enzyme that catalyzes the decomposition of hydrogen peroxide into water and oxygen, and is one of the important antioxidant enzymes that protect cells from reactive oxygen species. Supplementation of different concentrations of vitamin A in rats decreased the activity of CAT in the lungs, causing oxidative stress [23]. But our results showed that blood CAT levels increased significantly after the addition of vitamin A. In this study, vitamin A enhances the antioxidant capacity of weaned piglets by increasing CAT level.

In addition, vitamin A is also essential in humoral immunity, especially for antibody production [24]. Immunoglobulin is synthesized by plasma cells and lymphocytes. As an antibody, it is abundant in serum, accounting for about 20–25% of the total protein in serum. In addition, studies have shown that the addition of gelatin and vitamin A starch can improve the levels of IgA and IgM in serum [15]. IgM is the primary antibody of the primary immune response and the most common immunoglobulin expressed on the surface of B cells. The most prevalent antibody in blood circulation, IgG binds to a wide range of pathogens, including bacteria, fungi, and viruses, to prevent infection [25]. IgA is the main antibody heterotype produced on the mucosal surface, which can effectively prevent bacterial colonization and transfer in intestinal mucosa [26]. Our results showed that vitamin A did not significantly increase the serum immunoglobulin content of weaned piglets, but there was no downward trend. Our results may be related to the method of vitamin A supplementation.

The principal organ for nutrition digestion and absorption, the intestine also acts as a selective barrier against both endogenous and foreign antigens. Intestinal health, particularly the integrity of the intestinal barrier, is essential for preserving the organism’s healthy state. Tight junctions, which are the primary determinants of intestinal barrier function and close the paracellular space between adjacent epithelial cells, are necessary for intestinal integrity [27,28]. Vitamin A deficiency caused a colon-intestinal barrier in mice, which not only reduced the secretion of immunoglobulin A, but also down-regulated the colon-tight-junction-related protein *Occludin* and *Claudin-1* [29]. Our results showed that the expression of *Claudin-1*, *Occludin,* and *ZO-1* in the jejunum and the expression of *Claudin-1* and *ZO-1* in the ileum of weaned piglets increased significantly after vitamin A was added. Vitamin A plays a protective role in the intestinal tract of weaned piglets. *E. coli* is one of the important causes of diarrhea in piglets. The addition of probiotics can improve the decreased expression levels of *Occludin*, *Claudin-1,* and *ZO-1* in the intestinal tract infected by *E. coli* [30]. More confirmation that vitamin A enhances tight junction protein gene expression may be one of the reasons for improving diarrhea in weaned piglets.

Vitamin A has been shown to be associated with inflammation in animals, and intestinal inflammation in piglets is expressed at the genetic level by the interleukin gene [31,32]. Immunity in the organism is a complex process and is often divided into innate and acquired immunity. Vitamin A may be involved in the immune response associated with the intestinal mucosa through the regulation of the cytokines *IL-4* [33]. Chen et al. observed that ATRA increased the mRNA of the *IL-4* receptor alpha chain, and increased *IL-4*-induced phosphorylation of the signal transducer and activator of transcription 6 (STAT6) and mRNA expression of chloride channels [34]. *IL-5* regulates the acquired immune response and acts on target cells by binding to its specific *IL-5* receptor (*IL-5*R). *IL-5* activates JAK1/2-STAT1/2 and Ras/ERK channels to act on inflammation in the body [35]. In addition to this, *IL-5* can stimulate the secretion of IgM and IgG by staphylococcal A-activated B cells [36], and recent reports have found that *IL-5* can collaborate with *IL-21* to induce the proliferation of IgA cells [37]. *IL-4* and *IL-5* are cytokines produced by TH2 helper T cells and mast cells, basophils, and eosinophils, both markers of the development of TH2 inflammation, along with systemic inflammation [38]. Nutritional regulation of allergic inflammation of mucosal surfaces is of great importance. Vitamin A deficiency decreased the TH2 response and increased the TH1 response [39]. In this study, the addition of vitamin A significantly increased the expression of *IL-4, IL-5,* and *IL-10* in the gut. Vitamin A has a regulatory effect on stress-induced intestinal inflammation in weaned piglets, but its effect on related pathways needs to be confirmed by subsequent experiments.

The gut microbiome is increasingly recognized to play a role in regulating host growth performance, metabolism, diarrhea [40], and inflammatory responses [41]. Dietary nutrients are digested and absorbed in the foregut before undigested components and endogenous substances are fermented by the gut microbiota in the hindgut [42,43]. The establishment of a healthy, stable, and diverse intestinal flora is important for weaning piglets to resist stress and intestinal disease. In the present study, we demonstrated significant differences in the structure and content of intestinal flora between piglets fed vitamin A and control piglets. α diversity can be used as an indicator of the functional resilience of the intestinal microbial ecosystem, including species richness and species diversity [44]. From our experimental results, there was no significant change. PCA and PCoA are methods for downscaling and displaying multidimensional data differences on a two-dimensional coordinate plot. The closer the samples are in the graph, the more similar their community composition is [45]. Our data show that after the addition of vitamin A, the cecum flora of piglets still changed and did not completely overlap. The ratio of thick-walled to Bacteroidetes phylum is a common evaluation indicator that represents the imbalance in intestinal flora composition [46]. Our study discovered an increase in *Bacteroidetes*, and it was hypothesized that vitamin A altered the composition of weaned piglet cecum flora. *Actinobacteria* are Gram-positive bacteria with mycelium and substrates that are rich in bioactive secondary metabolites such as enzymes, antibiotics, and antioxidants [47]. *Actinobacteria* also produce a variety of antimicrobial agents [48]. In this experiment, we found that *Actinobacteria* at the gateway level increased the abundance of *Actinobacteria* in the piglet cecum. In one study [49], researchers discovered that *Erysipelotrichia* of the intestine reduces improving oxidative stress caused by weaning stress in piglets. Similar to our findings, it is hypothesized that vitamin A can reduce weaning-induced oxidative stress by decreasing *Erysipelotrichia*. Intestinal oxidative stress triggers intestinal microbiota dysbiosis, which is associated with post-weaning diarrhea and intestinal infections. Ellagic acid with antioxidant capacity can modulate the gut microbiota of weanling piglets to alleviate intestinal damage and oxidative stress [50]. We suspect that vitamin A has a similar effect on relieving diarrhea. Chai et al. [51] found from the transcriptome that the gene expression module for vitamin A effects in the small intestine differed in the colon, again perhaps because vitamin A absorption and metabolism occurs mostly in the small intestine. Although the small intestine and colon are contiguous organs with similar developmental origins, there are differences between their biological functions. The specific mechanisms by which vitamin A regulates the intestinal flora in weaned piglets are not fully defined by changes in flora abundance and need to be explored in more depth. In conclusion, 4400 IU/kg vitamin has positive effects on the growth performance, intestinal inflammation, and intestinal flora of weaned piglets.

## 5. Conclusions

Dietary supplementation with 4400 IU/kg vitamin A reduced the diarrhea rate of weaned piglets. Vitamin A improved weaned piglet catalase activity, improved weaned piglet antioxidant capacity, improved the intestinal barrier in the jejunum and ileum, regulated intestinal inflammation in the jejunum and ileum, and influenced cecum intestinal flora. This study may provide a foundation for the use of vitamin A in antibiotic-free piglet production, but more in vivo and ex vivo experiments are needed to prove this.

## Figures and Tables

**Table 1:** Basal diet composition and nutrient levels (feeding basis).

Ingredient ^1^	Content, %	Nutrition Levels ^2^	
Corn	40.57	DE kcal/kg	3598
Expanded corn	15.00	ME kcal/kg	3459
Fermented soybean meal	10.00	CP, %	20.06
Soybean meal	6.50	Ca, %	0.68
Fish meal	4.00	Total P, %	0.58
Whey powder	12.00	AP, %	0.41
Whey protein concentrate	5.00	SID Lys, %	1.54
Soybean oil	1.00	SID Met + Cys, %	0.88
Sucrose	2.00	SID Thr, %	0.94
50% choline chloride	0.20	SID Trp, %	0.26
Salt	0.35		
Calcium hydrogen phosphate, 2H_2_O	0.85		
L-Lysin hydrochloride	0.40		
DL-methionine	0.15		
L-threonine	0.08		
Stone powder-calcium	0.90		
Premix	1.00		
Total	100.00		

^1^ Provided per kilogram of diet: 660 IU vitamin D, 48 IU vitamin E, 1.5 mg vitamin K, 2 mg vitamin B_1_, 8 mg vitamin B_2_, 60 mg vitamin B_3_, 24 mg vitamin B_5_, 14 mg vitamin B_6_, 0.6 mg Folic acid, 0.04 mg vitamin B_12_, 0.16 mg biotin, 120 mg Fe, 70 mg Mn, 16 mg Cu, 0.7 mg I, 0.3 mg Se. ^2^ Values were calculated according to NRC (2012). ME, metabolic energy. CP, crude protein. AP, available phosphorous. SID, standardized ileal digestible.

**Table 2:** Primers used for real-time quantitative PCR analysis.

Gene	Forward Primer (5′-3′)	Reverse Primer (5′-3′)	Accession Number
*β-actin*	CCTGGCACCTAGCACAATGA	CCTGCTTGCTGATCCACATC	XM_003124280
*IL-4*	TACCAGCAACTTCGTCCAC	ATCGTCTTTAGCCTTTCCAA	NM_214123.1
*IL-10*	AGAGGGGTGTCTACAAAGCC	AGAGGTACAGCAGGGTTTCC	HQ026020.1
*IL-5*	GCTGAGCCAGACAAGACTCCT	TGAAATCATCAAGTTCCCATCGC	NM_214205.1
*Claudin-1*	GACTCCTTGCTGAATCTG	GCACCTCATCATCTTCCAT	AJ318102.1
*Occludin*	GCACCCAGCAACGACAT	CATAGACAGAATCCGAATCAC	XM_005672525
*ZO-1*	AGCCCGAGGCGTGTTT	GGTGGGAGGATGCTGTTG	XM_013993251

**Table 3:** Effect of diet supplementary Vitamin A on growth performance of weaned piglets.

Items	Dietary Supplementary Vitamin A, IU/kg						SEM	*p* Value
	0	1100	2200	4400	8800	17,600		
BW, kg								
0 d	8.28	8.25	8.44	8.14	8.11	8.20	0.10	0.954
12 d	12.53	12.19	12.38	12.42	12.34	12.43	0.12	0.986
28 d	22.13	22.10	21.61	22.65	22.49	22.40	0.22	0.816
ADFI, g/d								
0 to 12 d	482.64	472.22	472.22	468.75	468.75	493.06	7.88	0.950
13 to 28 d	863.98	883.46	786.48	896.30	932.50	919.38	15.02	0.106
0 to 28 d	700.55	707.22	651.80	713.07	733.75	736.67	11.53	0.323
ADG, g/d								
0 to 12 d	353.89	328.26	328.13	356.46	352.88	352.18	7.79	0.798
13 to 28 d	600.00	619.17	576.67	639.69	633.96	623.43	8.43	0.268
0 to 28 d	494.52	494.49	470.15	518.30	513.50	506.98	7.14	0.429
F:G, g/d:g/d								
0 to 12 d	1.37	1.45	1.46	1.32	1.34	1.40	0.02	0.128
13 to 28 d	1.44	1.42	1.36	1.40	1.47	1.48	0.01	0.100
0 to 28 d	1.42	1.42	1.39	1.37	1.43	1.45	0.01	0.278
Diarrhea rate, %								
0 to 12 d	9.03 ^b^	1.39 ^a^	1.39 ^a^	0.69 ^a^	2.78 ^a^	2.08 ^a^	0.34	0.000
13 to 28 d	25.52 ^b^	6.78 ^a^	14.06 ^a^	9.37 ^a^	10.42 ^a^	11.98 ^a^	0.73	0.000
0 to 28 d	18.45 ^a^	4.46 ^b^	8.63 ^b^	5.65 ^b^	7.14 ^b^	8.04 ^b^	0.39	0.000

^a,b^ Data were shown as means ± SEM (*n* = 12), different letters represented significant differences (*p* < 0.05). The same as below. ADG = (body weight at the end of the trial-body weight at the beginning of the trial)/days of the trial; ADFI = total feed intake during the experimental period/(experimental days × number of piglets); F:G = average daily gain/average daily feed intake.

## Data Availability

All experimental data and analytical results are included in this study. The raw 16S rRNA sequences have been deposited in the NCBI SRA under BioProject PRJNA879935. The datasets used or analyzed during the current study are available from the corresponding author on reasonable request.

## Supplementary Materials

The following supporting information can be downloaded at: https://www.mdpi.com/article/10.3390/antiox12122049/s1, Figure S1: Effect of vitamin A supplementation on serum antioxidant levels; Figure S2: Effect of vitamin A supplementation on the expression levels of tight junction proteins in the jejunum and ileum; Figure S3: Effect of vitamin A supplementation on the expression levels of inflammatory cytokines in the jejunum and ileum.

## Abbreviation

Vitamin A	Vitamin A
BW	Body weight
ADG	Average daily gain
ADFI	Average daily feed intake
F:G	Feed-to-gain ratio
GSH-Px	Glutathione peroxidase
CAT	Catalase
T-SOD	Total superoxide dismutase
T-AOC	Total antioxidation capability
IgG	Immunoglobulin G
IgM	Immunoglobulin M
IgA	Immunoglobulin A
*IL-4*	Interleukin 4
*IL-5*	Interleukin 5
*IL-10*	Interleukin 10
ZO-1	Zonula occludens-1
PCA	Principal component analysis
PCoA	Principal coordinates analysis
