# Supplementary material for: Effects of Vitamin A on Growth Performance, Antioxidants, Gut Inflammation, and Microbes in Weaned Piglets
Source: Antioxidants (Basel). 2023 Nov 27;12(12):2049. doi: 10.3390/antiox12122049 (PMC10740560; doi:10.3390/antiox12122049)
Supplement: Supplementary file 1 [file antioxidants-12-02049-s001.zip › antioxidants-2652179-supplementary.pdf]

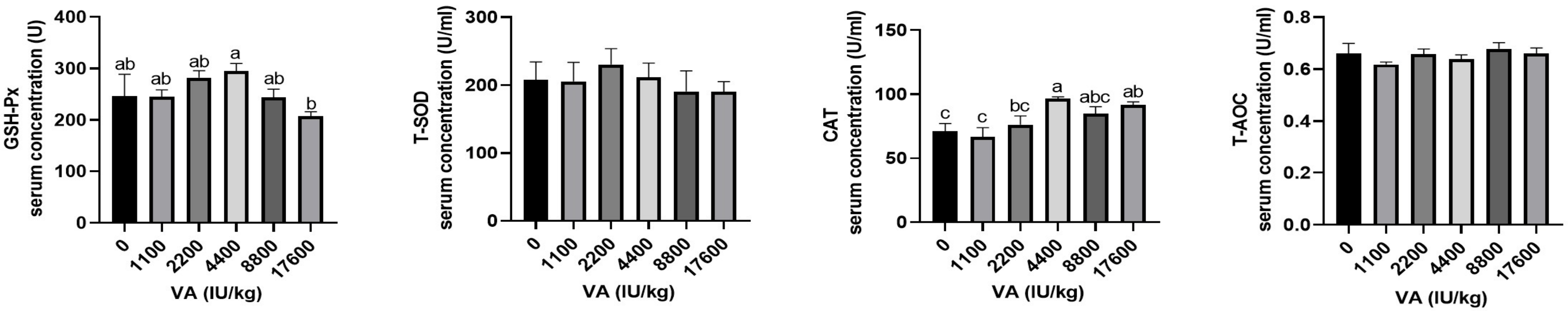

Figure S1. Effect of vitamin A supplementation on serum antioxidant levels. Data was shown as means ± SEM (n=6), different letters represent significant differences ($p < 0.05$).

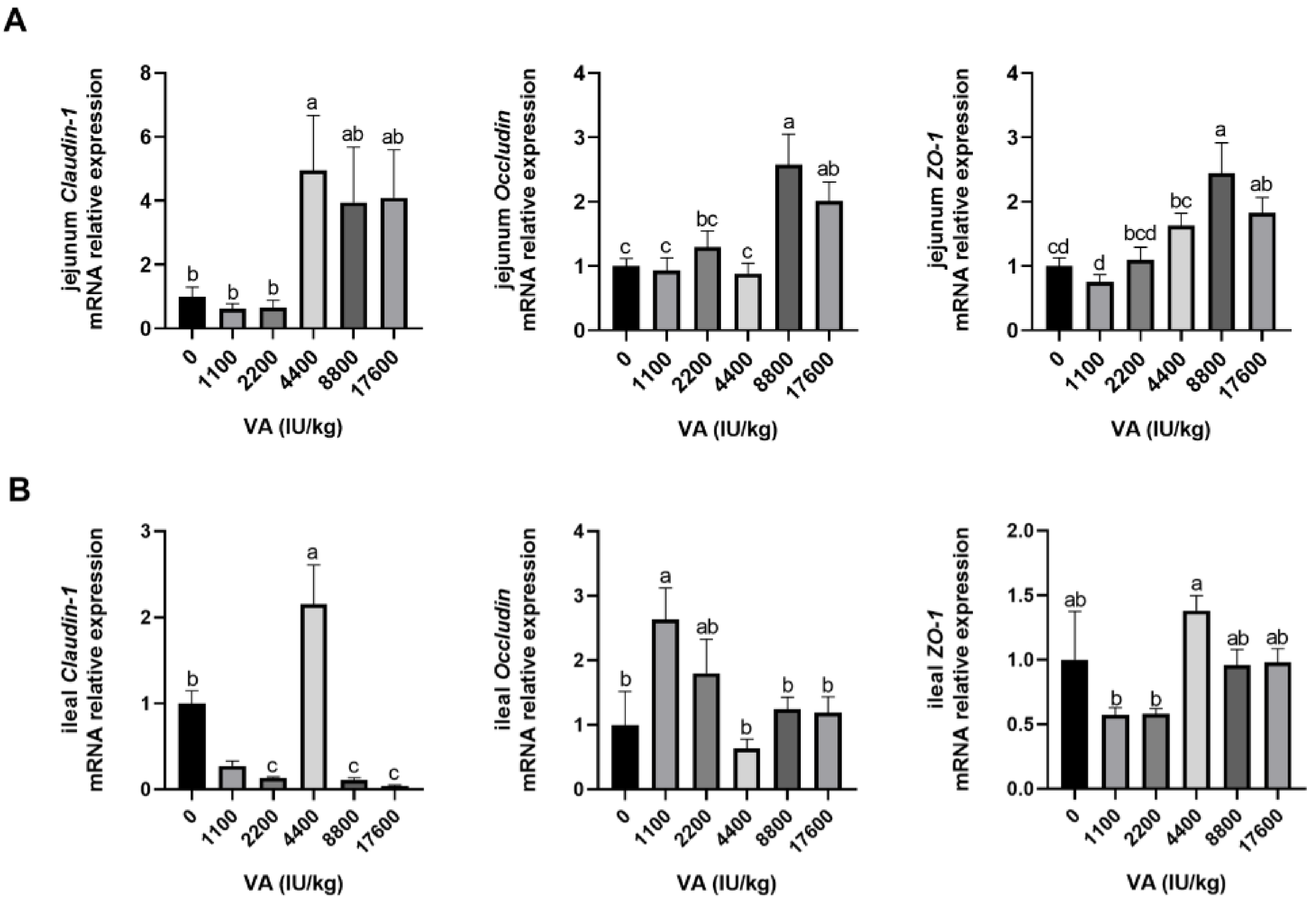

Figure S2. Effect of vitamin A supplementation on the expression levels of tight junction proteins in the jejunum (A) and ileum (B). Data was shown as means ± SEM (n=6), different letters represent significant differences ($p < 0.05$).

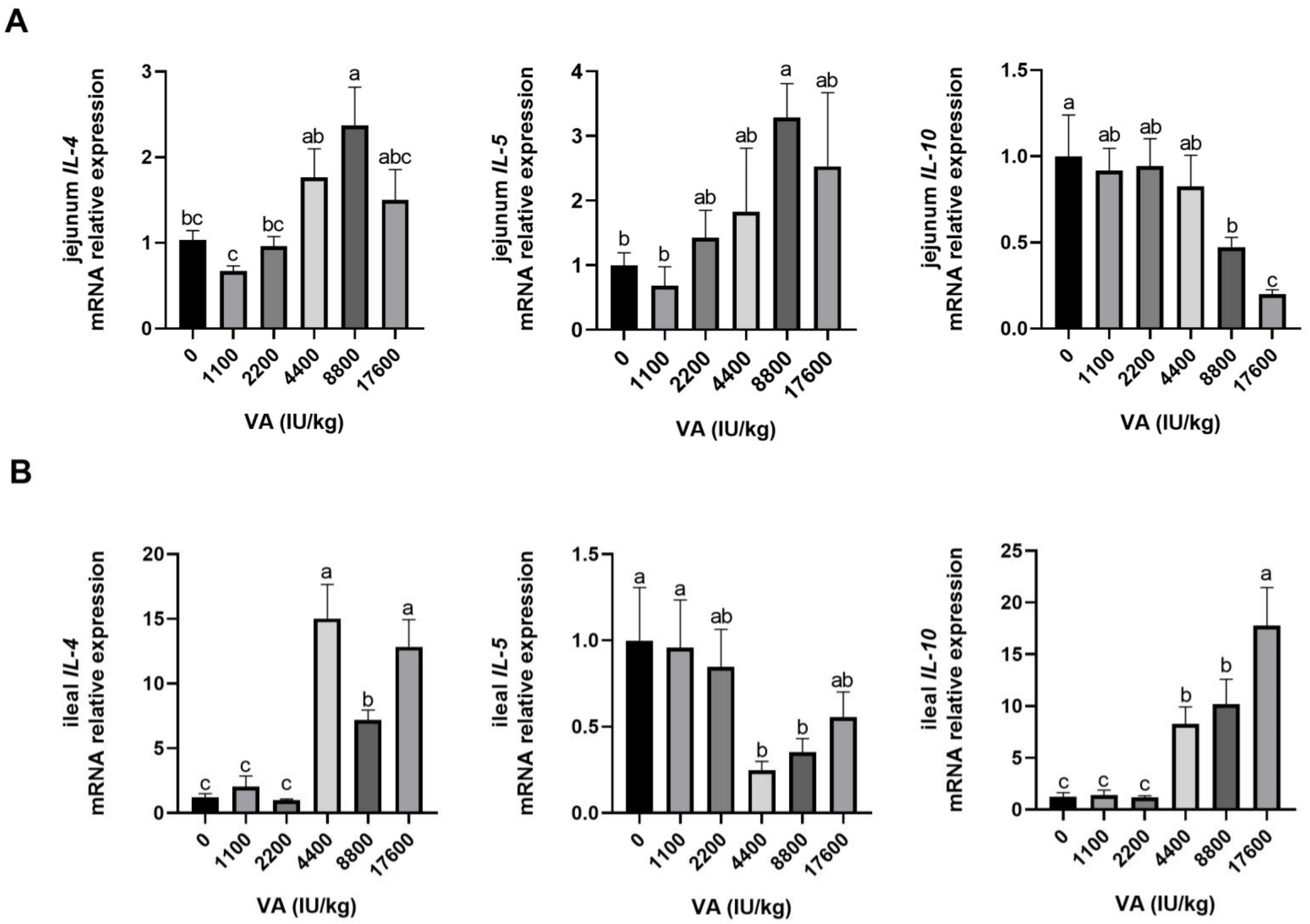

Figure S3. Effect of vitamin A supplementation on the expression levels of inflammatory cytokines in the jejunum (A) and ileum (B). Data was shown as means ± SEM (n=6), different letters represent significant differences ($p < 0.05$).
